# Hiding in plain smell

**DOI:** 10.7554/eLife.60912

**Published:** 2020-08-11

**Authors:** Youngsung Joo, Meredith C Schuman

**Affiliations:** 1 Department of Biology, Chungbuk National UniversityCheongjuRepublic of Korea; 2 Department of Geography, University of ZurichZürichSwitzerland; 3 Department of Chemistry, University of ZurichZurichSwitzerland

**Keywords:** oryza, nilaparvata lugens, chilo suppressalis, anagrus nilaparvatae, Other

## Abstract

A common rice pest can avoid its natural parasite by settling on plants that smell like they have been damaged by a species of caterpillar.

**Related research article** Hu X, Su S, Liu Q, Jiao Y, Peng Y, Li Y, Turlings TCJ. 2020. Caterpillar-induced volatiles provide enemy-free space for the offspring of the brown planthopper. *eLife* **9**:e55421. doi: 10.7554/eLife.55421

If you look carefully at a plant, you may start to notice a few telltale signs of feeding insects: holes chewed in a leaf, little mazes of trails, shiny spots of honeydew. You might even catch a caterpillar hiding along a leaf’s midvein – one of many strategies that plant-eating insects have evolved to camouflage themselves (Duncan, 1922). If you lean in, you will smell a planty scent, which herbivores use to choose the plants they eat (Bruce et al., 2005). At the same time, plants also release smells to attract species that prey on these herbivores (Turlings and Erb, 2018).

In fact, recent evidence suggests that plant odors are the subject of an ‘information arms race’, which plants seem to be winning so far. In this arms race, plants evolve new scents to become harder for herbivores to 'sniff out' in a crowd, while herbivores evolve to use more odors to find the plants they eat (Zu et al., 2020). In addition, plants also attract predators of herbivores, using smells that change depending on the herbivores feeding on the plant (Dicke and Baldwin, 2010). Together, these observations may explain why all plants studied so far produce rich, situation-dependent odor bouquets.

Now, in eLife, Yunhe Li from the Chinese Academy of Agricultural Sciences and colleagues from Switzerland and China – including Xiaoyun Hu as first author – report that a common crop pest can use the plant odors released by the feeding of another herbivore to hide from its own enemies (Figure 1; Hu et al., 2020). This strategy is known as ‘olfactory camouflage’ (Ruxton, 2009).

**Figure 1.**
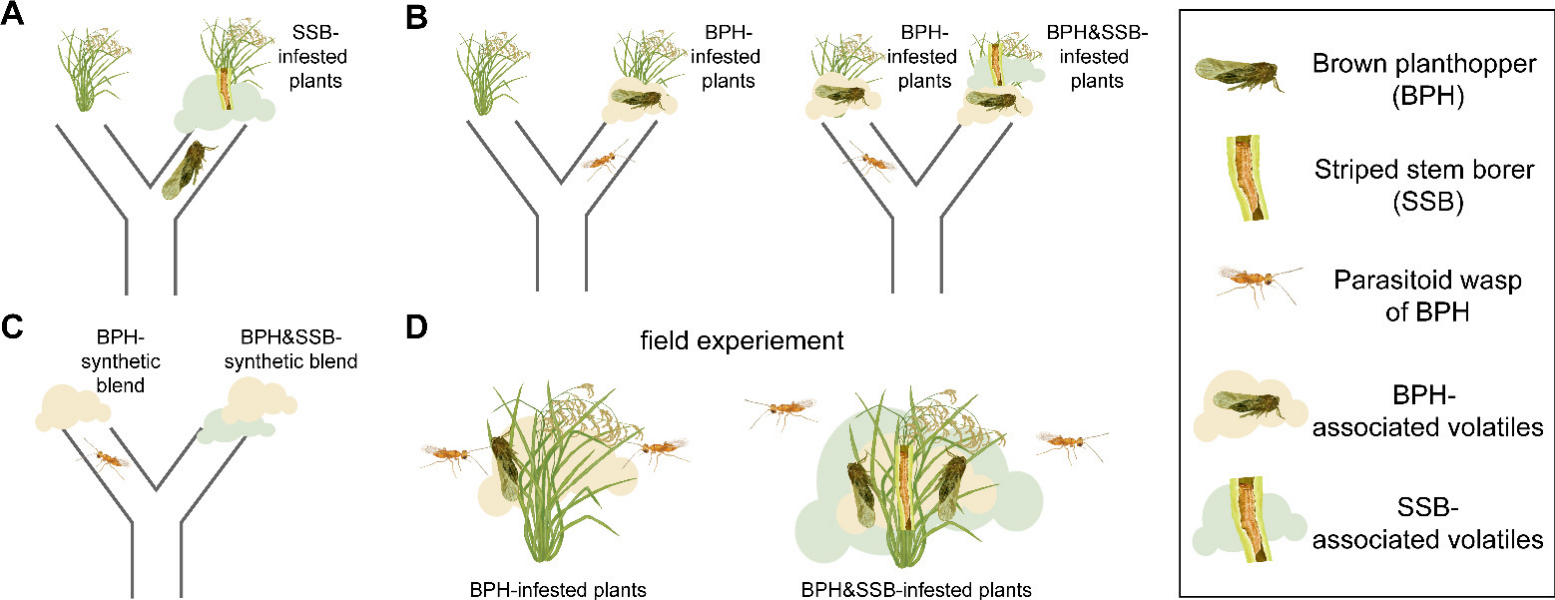
The level of infestation by striped stem borer caterpillars influences how brown planthoppers and the parasitoid wasp *Anagrus nilaparvatae* choose the rice plants on which to settle. (**A**) Brown planthoppers placed in the middle of a tube between an intact plant and a plant infested with striped stem borer caterpillars (SSB) usually choose the infested plant. This choice is likely based on the smell that the plant releases when being eaten by the caterpillars. (**B**) The parasitoid wasp *Anagrus nilaparvatae*, which lays its eggs inside planthopper eggs, prefers plants infested with ten brown planthoppers (BPH) over those infested with just five (left). However, if a striped stem borer caterpillar is added to the plant with ten brown planthoppers, the wasp has no significant preference for either plant. If a second caterpillar is added to the plant with ten brown planthoppers, the wasp then prefers the plant with five brown planthoppers (right). (**C**) Wasps placed in the middle of a tube between two synthetic scent blends never preferred the blends that smelled like striped stem borer caterpillars. (**D**) The results were consistent both in the wild and in the glasshouse, with wasps always preferring plants with brown planthoppers that were not infested with striped stem borer caterpillars.

Hu et al. focused on two widespread rice pests: the striped stem borer caterpillar and the brown planthopper. Rice can make different blends of odors to attract animals that rid the plant of feeding herbivores. One such animal is a species of wasp called *Anagrus nilaparvatae*, which lays its eggs inside planthopper eggs. Hu et al. first observed that brown planthoppers preferred to lay their eggs on caterpillar-infested rice plants rather than undamaged plants (Figure 1A). Next, experiments were performed to test whether *Anagrus nilaparvatae* wasps chose the plant on which to lay their eggs based on the presence of caterpillars. The results showed that, in the absence of caterpillars, wasps preferred plants with more planthoppers. However, when the plants had both planthoppers and caterpillars, the wasps instead preferred plants with fewer caterpillars (Figure 1B). This indicates that one or more odors emitted by the caterpillar-infested plants were masking the presence of planthoppers.

To test this hypothesis, Hu et al. identified 20 odor compounds whose levels varied depending on the densities of planthoppers and caterpillars on the plants. These compounds were then used to test which odors the wasps preferred. Finally, to test whether odor alone was sufficient to explain the wasps’ choice, Hu et al. made synthetic blends of 13 odors that affected the wasps’ behavior. This experiment had exactly the same results as using infested plants: wasps did not choose any odor blends that smelled like plants eaten by caterpillars, with or without planthoppers (Figure 1C). In fact, both in the glasshouse and in the field, wasps parasitize a smaller share of eggs on caterpillar-infested plants, even when the larger number of eggs on those plants is accounted for (Figure 1D). This corresponds to wasps’ preference for the odors of plants hosting only planthoppers, and not caterpillars.

Hu et al.’s results suggest that planthoppers currently have the advantage in their information arms race with rice plants and wasps. How did this happen? The blend of odors that rice produces to encode ‘caterpillar’ appears to be more complex than the blend for ‘planthopper’, so when both are present, information about the planthoppers may be lost. However, the information arms race model indicates that rice plants should evolve a counter-strategy (Zu et al., 2020); and indeed, Hu et al. further showed that olfactory camouflage is less effective in wild rice than in cultivated plants. Unlike wild rice, cultivated rice is under artificial selection pressure by humans and is not free to respond to natural selection.

These results indicate that reducing striped stem borer caterpillar infestations in rice can yield additional benefits, as it may promote biological control of the brown planthopper by parasitoid wasps. From an evolutionary perspective, however, this is shortsighted: if efforts to reduce caterpillar populations fail, planthoppers will continue using the caterpillars as camouflage unless rice plants and wasps evolve ways to elude this mechanism. Unfortunately, waiting for rice plants to evolve a response would entail several generations of reduced rice yield – a disaster for our food supply. An alternative may be to artificially select plants with an advantage in this evolutionary arms race. Doing so will first require dissecting exactly how the planthopper’s olfactory camouflage works, and better understanding how plant odors direct interactions between species.
